# A Split-Mouth Clinical Comparative Evaluation of the Anesthetic Efficacy of Articaine and Lignocaine for Maxillary Bicuspids Extraction

**DOI:** 10.7759/cureus.40167

**Published:** 2023-06-09

**Authors:** Pooja Jaiswal, Rohit Goyal, Chanda Dubey, Javed Akhter, Karthikeyan Ramalingam, Sangita Kalita

**Affiliations:** 1 Oral and Maxillofacial Surgery, Maharaja Ganga Singh Dental College and Research Centre, Sriganganagar, IND; 2 Oral Pathology and Microbiology, Saveetha Dental College and Hospitals, Saveetha Institute of Medical and Technical Sciences, Saveetha University, Chennai, IND

**Keywords:** orthodontic extraction, premolars, visual analog scale, pain, oral surgery, extraction, buccal infiltration, lignocaine, articaine, local anesthesia

## Abstract

Aim

The study compared the anesthetic effectiveness of articaine and lignocaine when premolars are extracted bilaterally for orthodontic purposes.

Material and methods

This prospective split-mouth study was performed on 30 cases selected from orthodontic referral patients reporting to the Oral and Maxillofacial Surgery Department at Maharaja Ganga Singh Dental College and Research Center, Rajasthan, India, for bilateral extraction of premolars under local anesthesia. We used 4% articaine hydrochloride and adrenaline 1:100000 (AH) as group A and 2% lignocaine hydrochloride with adrenaline 1:100000 (LH) on the control side as group B. For premolar anesthetization, 0.6-1.6 ml of AH and 1-2 ml of LH were injected submucosally in the buccal vestibular area. The extraction procedure was then carried out after achieving adequate anesthesia. The pain was assessed with Visual Analog Scale. The average onset time and duration of anesthesia were recorded. Data collected were summarized with descriptive statistics. The SPSS version 23.0 (IBM Corp., Armonk, New York) was used for data entry, validation, and analysis. Means of continuous variables were compared using the student t-test. All tests were 2-tailed and significant at equal or less than 0.05. (p≤0.05).

Results

When comparing the overall anesthetic efficiency, Group A had a lower overall pain score of 0.43 while Group B had a higher overall pain score of 2.9. The average onset time of anesthesia in Group A was 1.2 minutes and 2.55 minutes in Group B. In Group A, the average duration of anesthesia was 70 minutes, and 46.5 minutes in Group B. These parameters were statistically significant with a p-value of <0.05.

Conclusion

The study concluded that as an alternative to lignocaine, articaine could be used effectively for maxillary premolar extractions for orthodontic reasons obviating palatal injection which is very painful to the patient.

## Introduction

In dentistry, local anesthesia is crucial for pain management because it is thought to be the safest and most effective medication for pain prevention and management for patients undergoing the procedure of oral surgery. Local anesthesia provides the strength for pain management procedures. These are substances that block nerve conduction in a specific, entirely reversible, non-permanent manner without altering the patient’s consciousness [1,2].

Teeth are important for functions like speech, mastication, one’s personality, and even in facial esthetics. One of the most frequent procedures in the field of oral surgery is tooth extraction. Extraction of teeth can be done for many reasons like irreversible dental pulpitis, advanced periodontal diseases, and orthodontic purposes. Extraction of teeth is considered one of the most dreaded procedures by many people, due to the unpleasant past experience of previous tooth extraction done for them or for others [3-8]. There are many reported cases of routine tooth extractions in the literature. Extraction ought to be painless. Pain management intraoperatively in extraction procedures is of great importance [2].

The most commonly experienced symptom in dentistry is pain, and palatal injection is among the most unpleasant procedures. Since the injection is rather painful, numerous strategies have been proposed to lessen the patient’s agony. The most frequently used technique is topical anesthesia for atraumatic needle penetration. The palatal injection is painful due to the sensitivity of the palatal soft tissue and its tight attachment to the bone present beneath it [1,2].

A painful injection given palatally can be refrained by using the palatal diffusion of the local anesthetics with solitary buccal infiltration [8]. The maxillary bone’s composition varies depending on characteristics like age, gender, and race. When articaine is used as a buccal infiltration, the anterior section of the maxilla has a bone that is denser than the posterior area which can impact diffusion and anesthetic potential [3].

Articaine spread out into soft and hard tissues is more effective than other local anesthetics and its buccal infiltration in the maxilla provides soft tissue anesthesia on the palatal side, ruling out the necessity of uncomfortable palatal injection [4-8].

Hassan et al. [8] performed a study on articaine for orthodontic extraction of maxillary premolars at Yenepoya Dental College, Mangalore, which is in Southern India. They have also mentioned variations in perception and pain reaction among individuals, differences in techniques and regional anatomy, and reporting errors in anesthetic efficacy. They have also recommended controlled clinical trials and comparative studies to ascertain the effects of articaine.

Hence, we studied the efficacy of anesthesia with lignocaine and articaine for the extraction of bilateral premolars in orthodontic patients of Maharaja Ganga Singh Dental College and Research Center, Sriganganagar, Rajasthan, which is in North Western India. We analyzed the time for initiation of anesthesia, duration of anesthesia, and pain measurement with a Visual Analogue Scale (VAS) and also observed if there are any intra or post-administration complications.

## Materials and methods

This prospective split-mouth study was performed at Maharaja Ganga Singh Dental College and Research Center and was approved by the Institutional Ethics Committee (approval number MGSDC/54/21).

The safest and most efficient way to control discomfort and pain management during dental treatment is with local anesthetics. The study group comprised 30 individuals referred for bilateral extraction of maxillary premolars as a part of orthodontic treatment. The sample size calculation was performed using G-power based on the study by Hassan et al. [8]. The medical examination was performed to rule out pregnancy, hypertension, diabetes, and other immune deficiencies. Patients of both sexes of the age range 10-28 years without any systemic illnesses were included in the study. Informed consent was obtained before surgical procedures.

**Figure 1:**
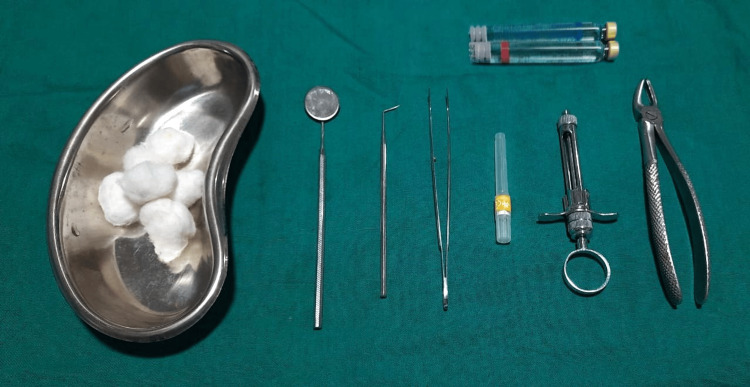
Armamentarium Picture showing the instruments utilised in this study

Blinding was not done. The choice of lignocaine or articaine was taken by the coin toss method. They were anesthetized either using Septodont Septanest: 1:100,000 - 4% articaine with epinephrine (Septanest, Septodont Inc, France) or Octocaine 100 - lidocaine HCl 2% and epinephrine 1:100,000 injection (lidocaine hydrochloride and epinephrine injection USP, Novocol, India), aspirating dental injection syringe of 1.8 ml capacity (GDC, India), disposable needles, 0.40 x 35mm (27 gauge) (Septoject; Septodont Inc, France), and routine extraction instruments (Figure 1).

On the dental chair, each patient was placed in a semireclined position. Patients were prepared and draped. Chlorhexidine mouthwash was used in all the patients prior to the procedure. The supraperiosteal approach involved pooling local anesthetic solution at the height of the mucobuccal fold above the tooth’s apex. It anesthetized tiny nerve terminals in the dental treatment area to avoid stimulation and impulse generation. 

**Figure 2:**
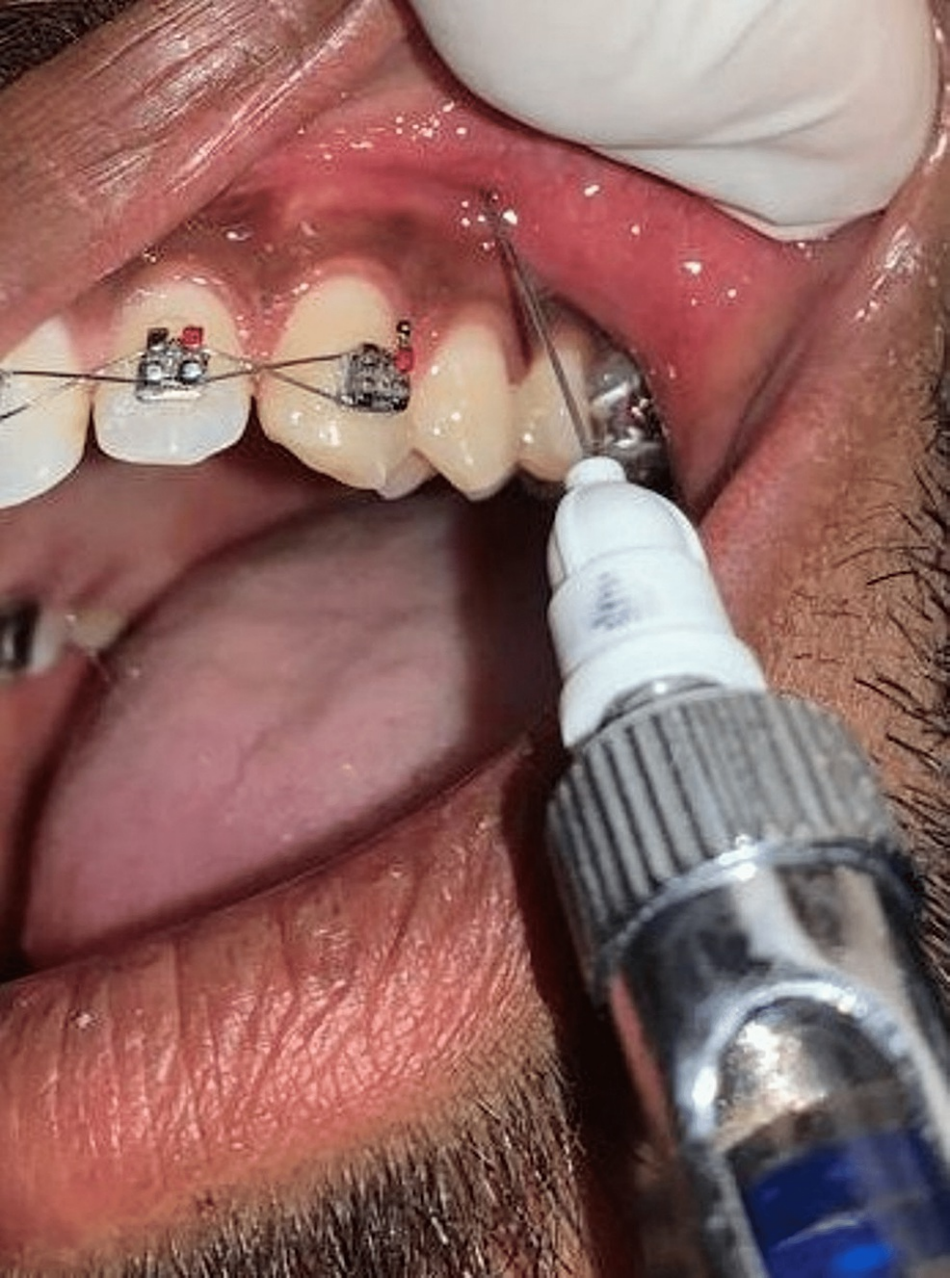
Clinical picture of articaine injection The clinical picture shows articaine infiltration on the buccal vestibule of the maxillary premolar region.

Orthodontic extraction of premolars was performed under aseptic conditions by a single operator. For anesthetizing the maxillary premolar region, 0.6 to 1.5 ml of 4% articaine was administered as a buccal, supraperiosteal injection (Figure 2).

Objective symptoms of anesthesia like the absence of pain were inspected on the palatal side. If anesthesia was achieved without additional infiltration, an extraction procedure was performed by elevation of mucoperiosteal soft tissues and utilization of premolar forceps.

**Figure 3:**
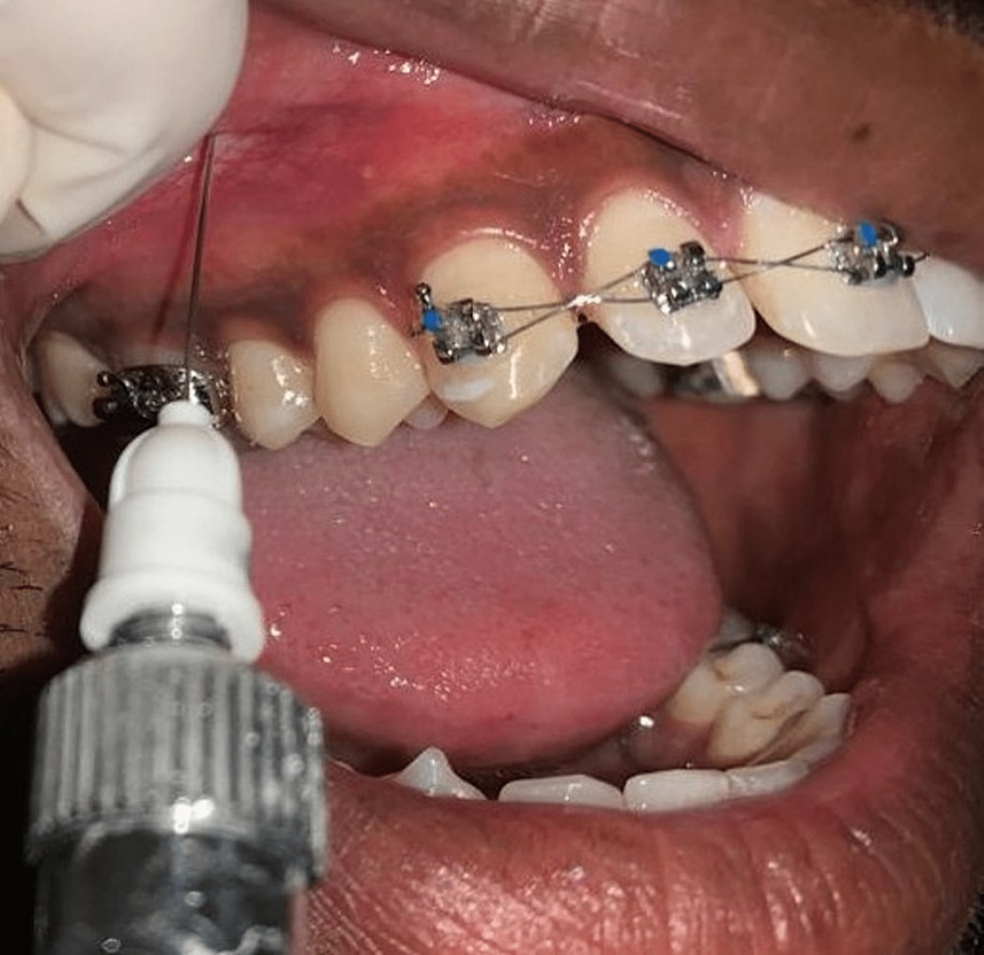
Clinical picture of lignocaine injection Clinical picture shows the lignocaine infiltration on the buccal vestibule of the maxillary premolar region.

On the opposite side of the same individual, 1-2 ml of 2% lignocaine was administered supraperiosteal on the buccal vestibule of the maxillary premolar region (Figure 3).

**Figure 4:**
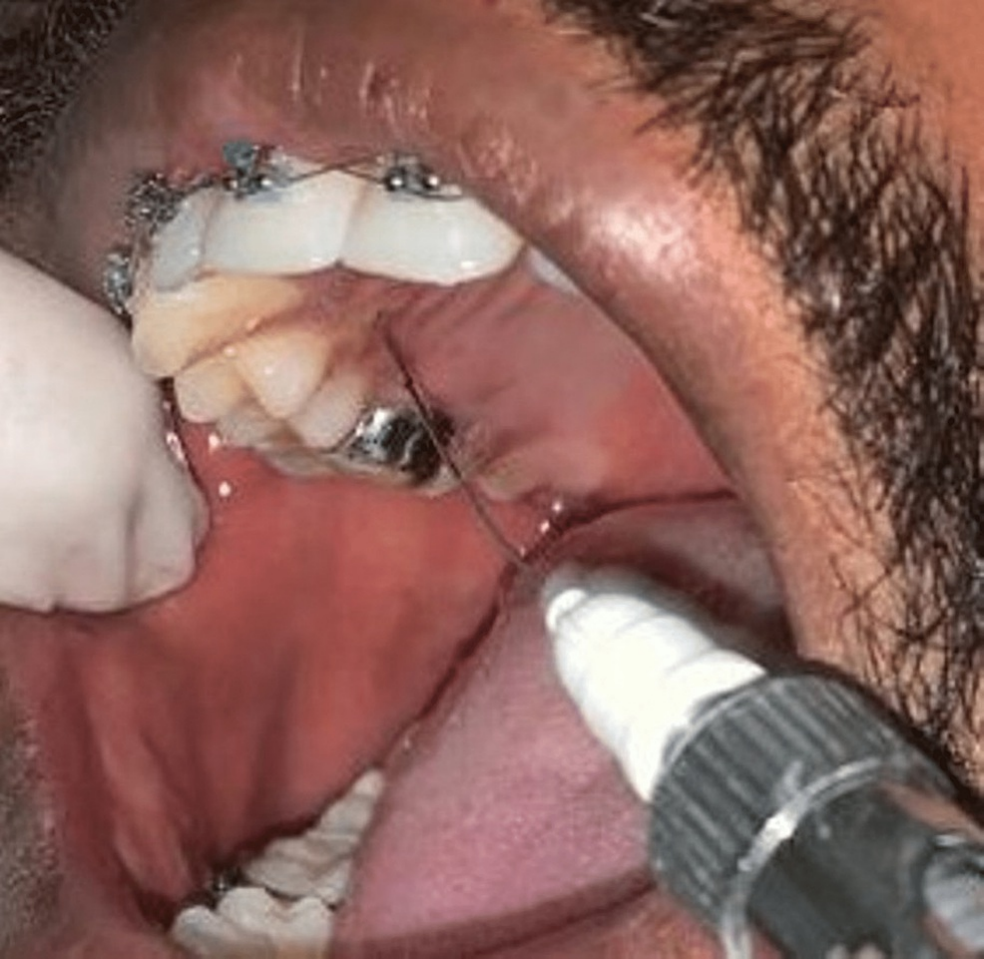
Clinical picture of palatal lignocaine injection Clinical picture showing the additional palatal infiltration of lignocaine

When objective symptoms like pain due to incomplete palatal anesthesia appeared, a supplementary infiltration on the palatal side was provided to anesthetize the palatal soft tissues prior to the extraction procedure, and the additional dosage of local anesthetic was recorded (Figure 4). The extraction process was performed after establishing full anesthesia.

Each patient was frequently interrogated regarding pain during the extraction process. Patients were assessed with a 100mm Visual Analogue Scale (VAS) ranging from 0 (no pain) to 100 (very severe pain). VAS was used to evaluate the amount of felt pain during the entire procedure and following the extraction. A pain VAS score of 30, 70, and 100 indicated the upper boundaries of mild, moderate, and severe pain intensity. The pain scores were divided by 100 and utilized for analysis.

Other study parameters were also evaluated by the single operator. The mean injection pain rating was measured using VAS. The average time for onset of anesthesia and average anesthesia duration was subjectively assessed by questioning the patient and confirmed with a clinical assessment with a rounded instrument. The patient was retained in the Oral Surgery department till the anesthesia wore off completely and was re-confirmed by subjective and objective assessment. The complications after injection were recorded and tabulated.

Statistical analysis was performed using SPSS version 23.0 (IBM Corp., Armonk, New York). Means of continuous variables were compared with the Student t-test. All tests were 2-tailed in nature and the significance level was set at equal or less than 0.05. (p ≤ 0.05).

## Results

This prospective split-mouth study was performed to assess the anesthetic efficacy of articaine and lignocaine on 30 individuals. Their age range was 14-26 years. Their mean age was 20 years and 47% of patients were above the mean age. Equal gender distribution was noted with 15 males and 15 females.

On the basis of malocclusion, we observed Class II Division 1 (n = 12), Class II Division 2 ( n = 5), Class I Bimaxillary protrusion (n = 9), and Class I with severe crowding (n = 4). All these individuals were referred for bilateral extraction of maxillary premolars. Articaine was administered in the A group and lignocaine was used in the B group.

A Visual Analog Scale was used to measure the pain during the procedure. On buccal infiltration, the pain score ranged between 30-50 for Group A and 40-60 for Group B. The mean injection pain rating buccally in Group A was found to be 0.4333 ± .8976, whereas, in Group B, it was noted as 0.5 ± 1.137. But, no statistically significant difference existed between the groups (P > .05). We also measured the VAS scores on the palatal mucosa after administering the anesthetic to the buccal vestibule. Group A had a lower VAS score than Group B after buccal infiltration. The pain score range on the palatal mucosa with only buccal infiltration was 0-10 for Group A and 50-70 for Group B. None of the Group A patients needed palatal infiltration, but all of the Group B patients needed additional palatal infiltration. The VAS score of supplemental palatal injection in Group B ranged between 60-80 and it was zero for Group A.

Hence, calculation of individual VAS scores for buccal infiltration, the efficiency of palatal anesthesia after buccal infiltration only, the VAS score for additional palatal infiltration in Group B, and overall anesthetic efficiency during extraction showed that Group A had a lower overall pain score of 0.4333 while Group B had a higher overall pain score of 2.9. The difference was highly significant statistically (P< 0.05).

**Figure 5:**
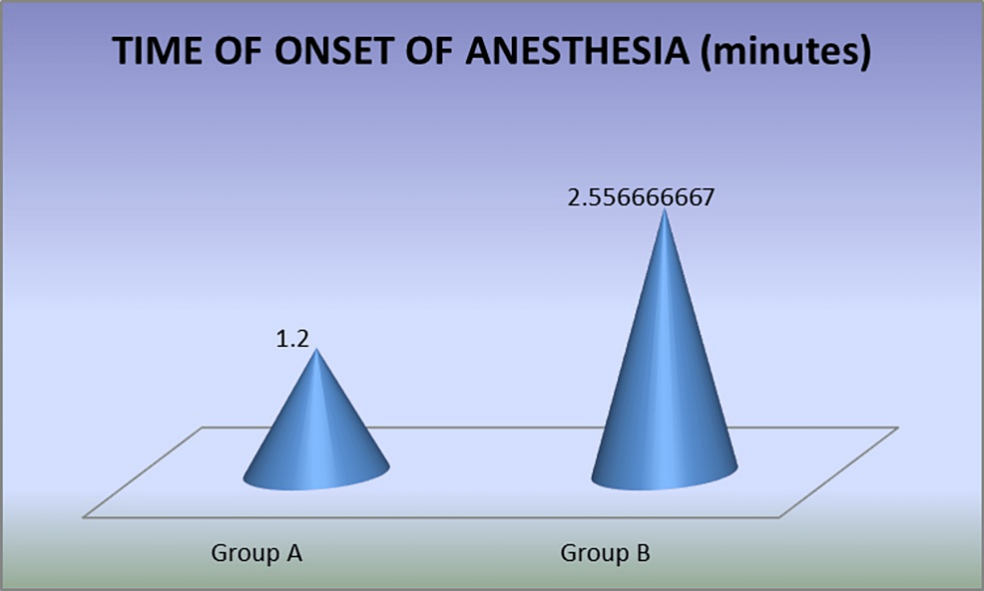
Graph representing time of onset of anesthesia Graph showing the quicker onset of anesthesia in the articaine group (Group A) compared to the lignocaine group (Group B).

The average time of onset of anesthesia in Group A was seen as 1.2 ± .46 minutes and 2.55 ± .69 in Group B. The difference was highly significant statistically (P < 0.05) between both groups. Group A experienced a quicker onset of anesthesia than Group B (Table 1, Figure 5).

**Table 1:** Table showing the time taken for onset of anesthesia Table showing the quicker onset of anesthesia in articaine group (Group A) than lignocaine group (Group B)

Parameter		N	Mean	SD	p-value
Onset of Anesthesia (minutes)	Group A	30	1.2	0.466092	0.039
	Group B	30	2.556667	0.692165	

The average anesthesia duration was 70 ± 14.38 minutes in the A group and 46.5 ± 4.57 minutes in the B group. The difference was highly significant statistically (P < 0.05) and showed that A group had a longer action duration compared to Group B (Table 2).

**Table 2:** Table showing duration of anesthesia Table showing that the mean duration of anesthesia was higher in the articaine group (Group A) than the lignocaine group (Group B).

Parameter		N	Mean	SD	p-value
Duration of Anesthesia (minutes)	Group A	30	70	14.3839	0.048
	Group B	30	46.5	4.576929	

In Group A, the mean Drug Volume used was 0.833± 0.212 ml, and 1.286 ± 0.293 ml in Group B. We also observed that none of the Group A patients needed a palatal infiltration, but all of the Group B patients underwent palatal infiltration.

In either the lignocaine or articaine groups, we did not observe any pain during the extraction procedure, nor were there any associated complications.

## Discussion

Dental pain management is widely performed with a local anesthetic agent. Because of its comparative safety and potency, articaine is a newly emerging local anesthetic agent [5]. Articaine is reported to be effective with a single buccal infiltration, and a separate palatal injection could be avoided [6-8]. Hence, we utilized 4% articaine with 1:100000 epinephrine to assess its efficacy in comparison to 2% lignocaine with epinephrine.

Robertson et al. [6] reported effective analgesia with articaine delivered by local infiltration for mandibular posterior teeth. Hassan et al. performed a study comparing articaine vs lignocaine for orthodontic extraction of maxillary premolars on 20 individuals, five males and 15 females, with a mean age of 21 years, but they did not specify the type of malocclusion [8]. Our study had 30 individuals of 15 males and 15 females with a mean age of 20 years. On the basis of malocclusion, we observed Class II Division 1 (n = 12), Class II Division 2 ( n = 5), Class I Bimaxillary protrusion (n = 9), and Class I with severe crowding (n = 4).

Studies have reported that articaine is effective at lower volumes than lignocaine, as it created a concentration of active anesthetic molecules at the injection site that is twice as high as lignocaine [4-6]. Sreekumar et al. [7] found that 1.2 milliliters of articaine with epinephrine for maxillary infiltration anesthesia had a quicker onset, higher success rate, and longer duration. This could be attributed to the increased lipid solubility of articaine that allows the anesthetic to pass even through thick cortical plates [4].

Hassan et al. stated that the onset period for articaine was 0.975 ± 0.1118 and lignocaine was 2.950 ± 0.5104 min, which showed a statistically significant difference [8]. In this study, mean anesthesia onset times in groups A and B were 1.2 ± 0.46 and 2.55 ± 0.69, respectively. The difference is highly significant statistically (P< 0.05) between both groups. Group A experienced the onset of anesthesia quicker than Group B. The inherent qualities of the drug substance that the anesthetic technique utilized influence when the effects manifest. Whereas, the corresponding pKa value has a direct impact on latency. A shorter delay is correlated with lower pKa values. Theoretically, 2% lidocaine (pKa = 7.9) would have a longer latency than 4% articaine (pKa= 7.8) [8].

Cowan A [5] reported that when articaine is used with a vasoconstrictor, it had a quick and prolonged action as it produced a high concentration of active anesthetic molecules locally. According to Kambalimath et al., the average length of pulpal analgesia with articaine infiltration was noted as 61.8 ± 59 minutes [9]. Mepivacaine anesthesia lasted 273.80 ± 15.94 minutes as reported by Colombini et al. [10]. Costa et al. reported 56.7 minutes of anesthesia for articaine [11]. In this study, in Group A, the average anesthetic duration was seen as 70 ± 14.38 minutes and 46.5 ± 4.57 minutes with Group B. The difference was significant statistically (P< 0.05) between the two groups revealing that group A’s activity lasted longer than group B’s. Our values are closer to those reported by Hassan et al. [8] with articaine’s anesthetic duration being 72 ± 17.275 minutes and lignocaine being 49 ± 5.026 minutes, which showed a statistically significant difference. This could be due to the inherent nature of the Indian ethnicity of the studied patients.

Group A had a lower overall pain score of 0.4333 while Group B had a higher overall pain score of 2.9 with a high statistical significance. We also found that articaine had quicker onset and longer duration of anesthesia along with enhanced patient compliance. This improved efficacy of articaine is also reported by other studies [11-18].

In comparison to 2.6ml ± 0.09 of lidocaine, Malamed et al. [16] found that the average amount of articaine needed to achieve anesthesia was 2.5 ± 0.07 ml for straightforward procedures like single extractions and 4.2ml ± 0.15 ml for more involved ones like multiple extractions, alveloectomies, and other osseous procedures [8,15]. According to Malamed et al. [16], when compared to both buccal and palatal injections of lidocaine, a single buccal infiltration of articaine produces better anesthesia for extracting premolars of the maxilla [17]. According to the reported literature, maxillary permanent teeth can be extracted with only buccal infiltration using articaine [1,2,18].

Adequate pain control has a pivotal role in dental practice [19]. Similarly, in our study, we substantiated that maxillary premolars could be extracted with only buccal infiltration of articaine to achieve adequate analgesia avoiding the need for palatal infiltration using lignocaine.

Limitations

The limitation of this study is the smaller sample size of 30 patients and the fact that only orthodontic extraction cases were taken into consideration. We also compared only the efficacy of maxillary anesthesia between articaine and lignocaine.

## Conclusions

Lignocaine is considered to be the industry standard for local anesthesia. Our research draws the conclusion that a replacement of lignocaine is possible with articaine which offered quicker onset, only buccal infiltration, longer duration of anesthesia, and better patient comfort with lower pain scores. Hence, It can be used for maxillary premolar extractions for reasons like in orthodontics which obviates painful palatal injection with additional efficacy and safety than lignocaine.

There was no difference in pain perception on the buccal side with both anesthetics. However, to support our findings, additional research is needed to analyze the efficacy of articaine in other local infiltration and nerve block techniques in the maxilla or mandible.
